# Left Ovarian Vein Thrombosis Presenting as Acute Postpartum Pyelonephritis

**DOI:** 10.7759/cureus.6854

**Published:** 2020-02-03

**Authors:** Erum Azhar, Truongson Nguyen, Abdul Waheed

**Affiliations:** 1 Obstetrics and Gynecology, Maimonides Medical Center, Brooklyn, USA; 2 Family Medicine, Wellspan Good Samaritan Hospital, Lebanon, USA

**Keywords:** ovarian vein thrombosis, post-partum fever, pyelonephritis

## Abstract

Ovarian Vein Thrombosis (OVT) is an extremely rare and uncommon thrombotic condition commonly attributed to the postpartum period. We report a case of a 30 yr old P2002 who presented with one day history of fever, chills, vomiting, abdominal and left flank pain. Patient had a preterm vaginal birth at 34 weeks gestation, four days prior to her presentation.

Patient was febrile on presentation with left CVA tenderness and diffuse abdominal tenderness. Pelvic Ultrasound showed enlarged uterus 14.7cm x 10.9cm x 8.5cm consistent with a postpartum uterus, with heterogeneous endometrium 2.3 cm, no retained products and normal adnexa. CT scan with contrast showed fluid along the anterior aspect of the left anterior kidney, left psoas muscle and extending down to the left side of the uterus and extending to the region of the left renal vein which confirmed left ovarian thrombosis. A CT Chest with contrast and bilateral lower extremity Doppler ruled out pulmonary embolism and deep vein thrombosis, respectively.The patient was admitted, treated with antibiotics and therapeutic dose of low molecular weight heparin (Enoxaparin) and responded well. Patient was discharged home on oral apixaban.

The clinical presentation of OVT is non-specific and can be similar to that of acute pyelonephritis. Physicians should have a high index of suspicion in postpartum patients presenting with flank pain and imaging techniques such as MRI, CT scan and ultrasound should be used to help in making the diagnosis.

## Introduction

Ovarian Vein Thrombosis (OVT) is a relatively rare and uncommon thrombotic condition commonly attributed to the postpartum period. It has been shown to complicate 1 in 600 to 1 in 1200 pregnancies [1]. It is more common after cesarean section than spontaneous vaginal delivery [2]. It is reported in gynecologic abdominal and pelvic surgery, malignancy, pelvic inflammatory disease and inflammatory bowel diseases and other conditions that can cause thrombus formation in the ovarian veins [3-5]. With the increased trend of laparoscopic gynecologic surgeries, OVT is also reported in patients who had undergone minimally invasive surgeries [6-7]. 

Clinical symptoms are vague and not very specific but most common presentation is in the puerperal period with fever and abdominal pain. However fatal pulmonary embolism has been reported in literature during cesarean section [8]. We report a rare case of ovarian vein thrombosis with presenting symptoms similar to acute left pyelonephritis four days after a spontaneous vaginal delivery of a preterm birth.

## Case presentation

A 30 years old P2002 postpartum patient presented to our Emergency department with one-day history of fever, chills, nausea, vomiting, abdominal and left flank pain. She had a precipitous preterm vaginal birth at 34 weeks gestation with an unknown GBS status, four days prior to presenting in the emergency department. The baby was found to have murky yellow vernix at the time of the delivery. The patient signed out against medical advice six hours postpartum. 

Review of placental pathology showed mild chorioamnionitis and three vessel cord. Patient’s past medical history was significant for epilepsy and had a last seizure in 2016. Her past surgical history was unremarkable. She was a smoker and had smoked throughout the pregnancy despite counselling on cessation of smoking. She stated she had allergy to Augmentin and that she develops a rash. 

In the emergency department, the patient was febrile (100.4 °F), with tachycardia and saturating 98% on room air. Her physical examination was remarkable for poor hygiene and dentition. She had diffuse abdominal tenderness, no abdominal distention, no rebound or guarding present, however had severe left CVA tenderness. Pelvic examination was remarkable for severe anterior vaginal wall tenderness; however, no foul-smelling discharge or uterine tenderness was present. 

**Figure 1:**
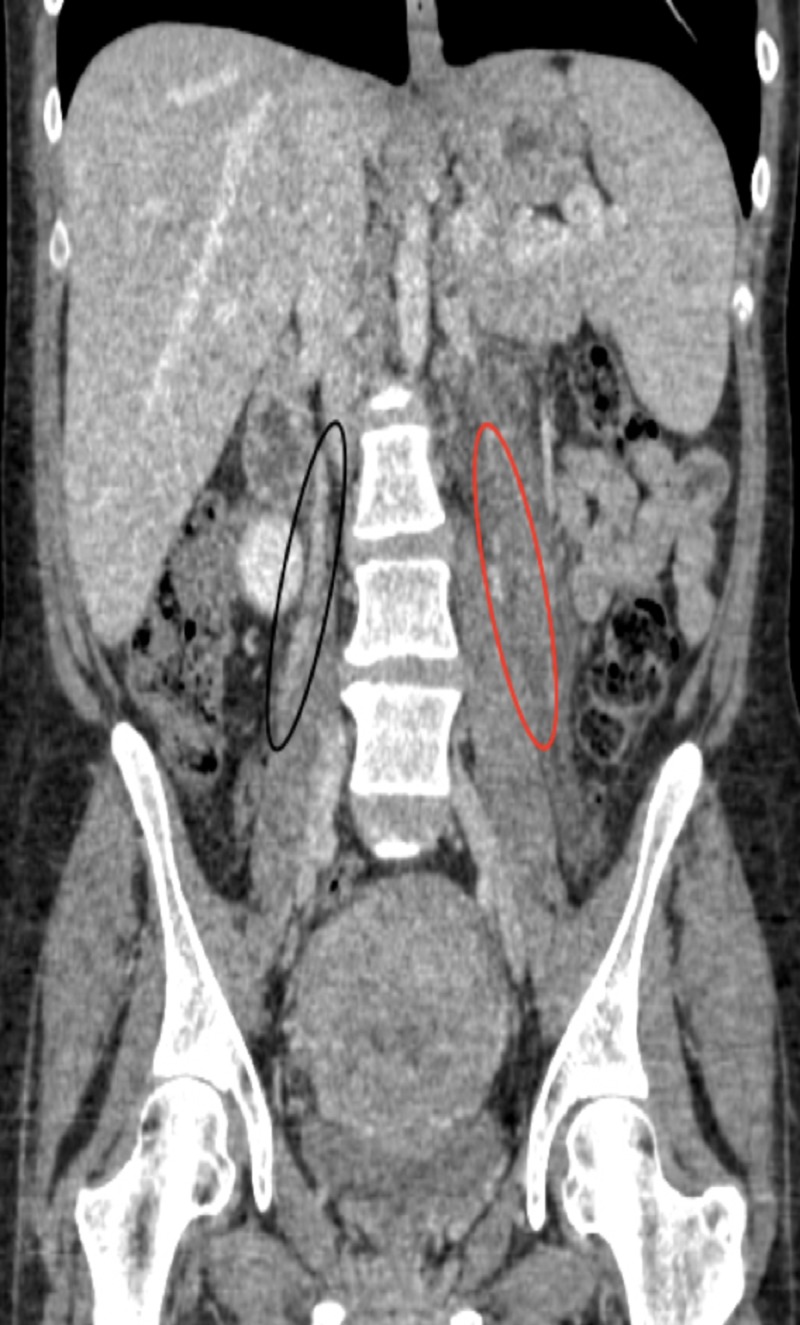
CT scan Coronal contrast-enhanced CT image demonstrates enlargement of Left gonadal/ovarian vein (RED) with central hypo-density and enhancement of vessel wall. Inflammatory changes in adjacent retroperitoneal fat can also be seen. Normal contrast flow through right gonadal vein is shown in (Black).

**Figure 2:**
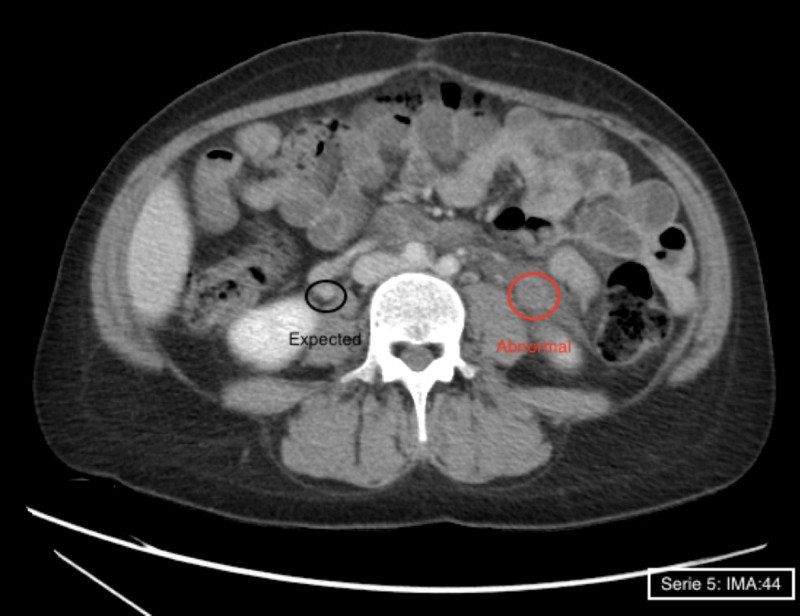
CT Scan Axial contrast-enhanced CT image demonstrate enlargement of Left gonadal/ovarian vein (RED) with central hypo-density and enhancement of vessel wall. Inflammatory changes in adjacent retroperitoneal fat can also be seen. Normal contrast flow in the Right gonadal vein is shown in (Black).

Her labs were significant for iron deficiency anemia, negative urinalysis, and D dimers were 3.34 (normal range 0.00-0.49 mg/L FEU). Blood and urine cultures were negative. Pelvic ultrasound was performed to rule out retained products that showed enlarged uterus 14.7cmx10.9cmx8.5cm consistent with a postpartum uterus, with heterogeneous endometrium2.3 cm, normal adnexa and no free fluid. A CT scan with contrast was obtained in view of the excruciating pain that showed fluid along the anterior aspect pf the left anterior kidney, left psoas muscle and extending down to the left side of the uterus and extending to the region of the left renal vein which confirmed left OVT (Figures 1, 2). Considering the diagnosis of the left OVT, a CT chest with contrast to rule out concomitant pulmonary embolism and a bilateral lower extremity doppler to rule out concomitant deep vein thrombosis were done which were both negative, respectively. As the patient presented with abdominal pain, fever and severe left CVA tenderness, the differential diagnosis at presentation was very extensive including gynecological and non-gynecological causes of pain including but not limited to renal colic due to nephrolithiasis, pyelonephritis, left ovarian torsion, tubo-ovarian abscess, endometritis with retained products, appendicitis and musculoskeletal pain. Imaging modality especially CT scan helped in definitive diagnosis of OVT.

Patient was admitted for IV antibiotics (Vancomycin, metronidazole and ciprofloxacin due to Augmentin allergy), and subcutaneous therapeutic enoxaparin anticoagulation.

The patient responded well. Within 24 hours of starting therapeutic anticoagulation, the patient became afebrile and her leukocytosis improved. She was discharged home on oral anticoagulant (Eliquis) on hospital day 2 after being afebrile for 48 hours.

## Discussion

OVT is a rare (fortunately) thrombotic but a serious postpartum complication. Because of the non-specific presentation, an increased index of suspicion is required as the potential for complication is high.

The ovarian vein drains directly into the inferior vena cava on the right, whereas on left side, it drains into renal vein. About 90% of the cases reported are unilateral and right sided. Some authors proposed a retrograde flow late in pregnancy in the left ovarian vein that might be protective against ascending infection, though this was not the case with our patient, since her thrombus was on left side [9]. The pathophysiology is identified as venous stasis, hyper-coagulability and endothelial damage that is caused by uterine infection during postpartum period [10]. A high index of suspicion for OVT in puerperal patients should be suspected especially those with nonspecific pain and fever not responding to conventional antibiotics. 

Previous studies have been inconclusive regarding the treatment of OVT. Plastini et al found that in patients with OVT treated with anticoagulation and in those without treatment there was no correlation found in terms of overall outcome [11]. However, a recent case-control study by Lenz CJ et al looked at the risk of venous thromboembolism recurrence, major bleeding and mortality among those who got diagnosed with ovarian vein thrombosis and found that although patients with OVT are treated less often (54%) as compared to patients who got diagnosed with lower extremity DVT (98%), the recurrence rate of venous thromboembolism was similar between these two groups (2.3% in OVT vs 1.8 in DVT ) arguing that anticoagulation with a direct oral anticoagulation or vitamin K antagonist should be considered especially in patients who have a personal history of venous thromboembolism [1]. We choose to administer the patient with anticoagulant and she did respond well. 

Further studies need to be done to look at the optimal duration of the treatment of anticoagulation in these patients and the antepartum management for the patients who get pregnant with a prior history of OVT.

## Conclusions

The clinical presentation of OVT is non-specific and can be very similar to that of acute pyelonephritis and should always be included in the differential diagnosis of left lower quadrant /flank pain in the postpartum patient. Physicians should have a high index of suspicion and imaging techniques such as MRI, CT scan and ultrasound should be used to help in making the diagnosis. Our patient had left OVT and CT scan with contrast helped in diagnosing the occluded vessel lumen as a low-density area indicating thrombus. It is wise to do CT chest to rule out pulmonary embolism in these patients as it is a known complication of OVT. Further studies also need to address the optimal duration of the treatment of anticoagulation in these patients and the antepartum management for those who get pregnant with a prior history of OVT.
